# Influence of Farming System and Forecrops of Spring Wheat on Protein Content in the Grain and the Physicochemical Properties of Unsonicated and Sonicated Gluten

**DOI:** 10.3390/molecules27123926

**Published:** 2022-06-19

**Authors:** Marta Tomczyńska-Mleko, Cezary Andrzej Kwiatkowski, Elżbieta Harasim, Justyna Leśniowska-Nowak, Stanisław Mleko, Konrad Terpiłowski, Salvador Pérez-Huertas, Olimpia Klikocka-Wiśniewska

**Affiliations:** 1 Institute of Plant Genetics, Breeding and Biotechnology, University of Life Sciences in Lublin, 20-950 Lublin, Poland; martamleko@tlen.pl (M.T.-M.); justyna.nowak@up.lublin.pl (J.L.-N.); 2 Department of Herbology and Plant Cultivation Techniques, University of Life Sciences in Lublin, 20-950 Lublin, Poland; czarkw@poczta.onet.pl (C.A.K.); o.klikocka@gmail.com (O.K.-W.); 3 Department of Dairy Technology and Functional Food, University of Life Sciences in Lublin, 20-704 Lublin, Poland; dairywhey@tlen.pl; 4 Department of Interfacial Phenomena, Maria Curie Skłodowska University, 20-031 Lublin, Poland; konrad.terpilowski@mail.umcs.pl; 5 Department of Chemical Engineering, University of Granada, Calle Prof. Vicente Callao, 3, 18011 Granada, Spain; shuertas@ujaen.es

**Keywords:** protein, wheat, farming system, forecrop, gluten, sonication, rheology, surface properties

## Abstract

The potential for enhancing the spring wheat protein content by different cultivation strategies was explored. The influence of ultrasound on the surface and rheological properties of wheat-gluten was also studied. Spring wheat was cultivated over the period of 2018–2020 using two farming systems (conventional and organic) and five forecrops (sugar beet, spring barley, red clover, winter wheat, or oat). The obtained gluten was sonicated using the ultrasonic scrubber. For all organically grown wheat, the protein content was higher than for the conventional one. There was no correlation between the rheological properties of gluten and the protein content in the grain. Gluten derived from organically grown wheat was more elastic than those derived from the conventional one. Sonication enhanced the elasticity of gluten. The sonication effect was influenced by the forecrops. The most elastic gluten after sonication was found for organic barley and sugar beet. The lowest values of tan (delta) were noted for conventional wheat and conventional oat. Cultivation in the monoculture gave gluten with a smaller susceptibility to increase elasticity after sonic treatment. Sonication promoted the cross-linking of protein molecules and induced a more hydrophobic character, which was confirmed by an increment in contact angles (CAs). Most of the organically grown wheat samples showed a lower CA than the conventional ones, which indicated a less hydrophobic character. The gluten surface became rougher with the sonication, regardless of the farming system and applied forecrops. Sonication treatment of gluten proteins rearranged the intermolecular linkages, especially disulfide and hydrophobic bonds, leading to changes in their surface morphology.

## 1. Introduction

Organic farming systems embrace environmental concerns and hope for the better nutritional value of the crops. Sustainable agricultural development is aiming at increasing the production of plant proteins. Crops obtained by agriculture are processed by the food processing industry. One of the basic ingredients of human food is flour and its functionality shaping ingredient—gluten. Important industrial applications of gluten make this substrate more and more attractive. In our previous research, we obtained gluten-based biopolymers with a possible application as matrices for active ingredients release or biodegradable pottery [1,2]

There are several research papers dealing with the influence of wheat cultivation forecrops on the protein and gluten content. Some of them are contradictory, which is probably caused by the complexity of the cultivation process. Thomsen et al. [3] noticed that protein is generally much less affected by forecrops and catch crops than grain yield. Jankowski et al. [4] found that the choice of forecrops had no significant influence on the protein content of wheat grain. The quality of the protein complex, including gluten content, was higher in the grain of wheat grown after oil plants than in the grain of winter wheat grown in monoculture.

Some researchers compared two farming systems: conventional and organic. Augspole et al. [5] found that organic winter wheat grains had a lower gluten content and water binding capacity, while gluten was significantly stronger in comparison to the conventional cultivation method. Krejcirova et al. [6] found that wheat varieties from conventional growing had twice the content of High-Molecular-Weight (HMW) glutenins in comparison to organic. HMW glutenins are responsible for dough elasticity. Wheat varieties from organic growing were characterized by a significantly higher percentage of nutritionally valuable albumins and globulins. In both systems of farming, the highest percentage of HMW glutenins was found in varieties from quality group E (elite, the most suitable for bread-making), while the varieties from quality group C (wheat unsuitable for bread-making) reached the highest percentage of residual albumins and globulins. 

A relatively new application within the food industry is power ultrasound for the modification of food microstructures. Ultrasound treatment (low frequency and high power) of foodstuffs generates regions of high hydrodynamic shear, elevated temperatures, and the potential for chemical reactions from free radical generation [7]. Ultrasound is an acoustic wave above the threshold of human auditory perception (>16 kHz). Acoustic waves are the propagation of mechanical (i.e., acoustic) waves of pressure and displacement through a medium, as longitudinal waves, exhibiting compressions (high-pressure regions) and rarefactions (low-pressure regions). Longitudinal waves are waves whereby the displacement of the medium is in the same direction as the wave [8]. Low-frequency (20–100 kHz), high-power-intensity (10–1000 Wcm^−2^) ultrasound is employed for the physical and chemical alteration, and generation and modification of foods [9]. The effects of power ultrasound on food structures are attributed to ultrasonic cavitation, the rapid formation and collapse of gas bubbles generated by localized pressure differentials (~50 MPa) occurring over short periods of times (a few microseconds). These ultrasonic cavitations cause localized regions of intense hydrodynamic shear forces and a rise in temperature at the site of bubble collapse (up to 5000 °C), contributing to the observed effects of power ultrasound [10].

There were some studies conducted on the influence of ultrasound on gluten, but all of them were performed on gluten dispersions in water. In those studies, sonicated gluten was centrifuged and freeze-dried and the physicochemical properties were analyzed. The obtained results are usually dependent on the duration and power of applied ultrasound. For instance, Zhang et al. [11] showed that as the pretreatment duration or power increases, the surface roughness, Young’s modulus, and adhesion of gluten also increase and then decrease. To the best of our knowledge, there are no results on the direct application of ultrasound waves on gluten.

The content of the basic nutrient in the grain—protein—is very important from a nutritional and technological point of view. Publications on the influence of the farming system on the grain protein content are contradictory and there are no publications on the influence of forecrops in different farming systems. There is no publication on the influence of these factors on the susceptibility of gluten to different processing methods. Thus far, researchers have been interested only in baking properties [12,13]. The challenge of conducting such research is the fact that it requires an analysis of the results from at least three years of cultivation. This increases the complexity and time of research. The aim of this research is to investigate the influence of the farming system and forecrops of spring wheat cultivation on the susceptibility of gluten to ultrasonic treatment. This study potentially opens a new chapter of research dealing with the influence of cultivation conditions on the susceptibility of gluten to different industrial processing. 

## 2. Materials and Methods

### 2.1. Field Experiment

A field experiment in growing spring wheat (*Triticum aestivum* L.—cv. ‘Monsun’) under organic and conventional farming systems was conducted over the period of 2018–2020 at the Czesławice Experimental Farm (51°30′ N; 22°26′ E; Lubelskie Voivodeship, Poland). The experiment was set-up as a split-plot design in 3 replicates of plots with an area of 80 m^2^ (8 m × 10 m). The total area of the experiment (24 plots) was 1920 m^2^. It was located on a loess-derived Luvisol, with the grain size distribution of silt loam (PWsp), classified as a good wheat soil complex (soil class II). Before the establishment of the experiment (2017, autumn), the soil was characterized by a medium content of available macronutrients (Table 1).

The experiment included:

I. Two farming systems of spring wheat:

CS (conventional system)—the recommended rates of mineral NPK (ammonium nitrate—34% N, enriched superphosphate—40% P_2_O_5_, and potassium chloride—60% K_2_O), seed dressing, fungicide and herbicide application, and mechanical weed control (harrowing before emergence and at the 3–4 leaf stage);

OS (organic system)—mineral fertilization with the fertilizer Humac Agro and mechanical weed control (harrowing before emergence and at the 3–4 leaf stage).

II. Five spring wheat forecrops:

Sugar beet (*Beta vulgaris* L. subsp. *Vulgaris*)—cv. ‘Everest’;

Spring barley (*Hordeum vulgare* L.)—cv. ‘Jovita’;

Red clover (*Trifolium pratense* L.)—cv. ‘Nike’;

Winter wheat (*Triticum aestivum* L.)—cv. ‘Bonanza’;

Oat (*Avena sativa* L.)—cv. ‘Agent’.

From 2015 (three years before the establishment of the experiment), the field with the organic system was managed following organic farming principles—a buffer zone (200 m) from conventionally farmed fields and no application of pesticides and artificial fertilizers (in 2015, the field received the Organic Farming Certificate awarded by the company ‘Eco-guarantee’). The distance of the experimental plots from the nearest traffic artery was 900 m.

In the conventional farming treatment, mineral fertilization is shown in Table 2:

In the case of the organic cropping system, the fertilizer Humac Agro was applied at the following rate: 350 kg ha^−1^ (before sowing). The chemical composition of the fertilizer Humac Agro is as follows: humic acid content: 62% on a dry weight basis; macro- and micronutrient content on a dry weight basis: N = 10.3 g kg^−1^, P = 1.05 g kg^−1^, K = 1.18 g kg^−1^, Ca = 16.80 g kg^−1^, Na = 12.80 g kg^−1^, Fe = 14.50 g kg^−1^, Zn = 64 mg kg^−1^, Br = 77 mg kg^−1^, Cu = 19 mg kg^−1^, and Se = 6 mg kg^−1^; moisture content: 20%.

Crop management operations in individual organically grown crops involved mechanical triple harrowing. Under the conventional system, crop management operations included the use of chemical crop protection products (seed dressing, herbicides, fungicides, insecticides, and retardants) from the product assortment and at the times and rates compliant with the Crop Protection Calendar of the Institute of Plant Protection—State Research Institute in Poznań [14], as well as the use of mechanical weed control identical to that used in the organic treatment (but harrowing operations in cereal crops were carried out twice, not three times as in the organic treatment). 

Chemical protection of spring wheat crops was carried out in the conventional system: seed dressing—Vitavax 200 FS (a.i. carboxin—200 g L^−1^, thiram—200 g L^−1^)—300 mL of 100 kg^−1^ of grain; herbicide—Sekator 6,25 WG (a.i. amidosulfuron + iodosulfuron-methyl-sodium + mefenpyr-diethyl)—0.2 kg ha^−1^; fungicide—Amistar 250 SC (a.i. azoxystrobin 250 g L^−1^)—0.6 L ha^−1^.

Tillage was typical for each plant species (spring wheat and spring wheat forecrops). 

During the research period, the date of sowing and harvesting of spring wheat was identical in the conventional and organic systems (it was in the range of 17–19 April —sowing, 18–20 August —harvest). The amount of spring wheat sowing was identical in the conventional and organic systems and amounted to 200 kg ha^−1^. 

The dates of sowing and harvesting spring wheat forecrops were also the same in both farming systems and were as follows (Table 3):

The fertilization of spring wheat forecrops was consistent with the farming system (conventional and organic) and fertilization recommendations for individual species. Detailed information on this subject is presented in Table 4 and Table 5:

After the spring wheat harvest, the grain was dried and brought to a moisture content of about 12–13%, and then grain samples were taken from each experimental variant for laboratory analysis. Each year, the grain protein content and gluten properties were analyzed. The presented results are average values with the standard deviation calculated for the results from different years. The nitrogen concentration in the grain was determined by the Kjeldahl method and the protein concentration was calculated by multiplying the nitrogen content by the conversion factor of 5.7 [15]. Twenty different combinations of the factors [forecrops (5) × cultivation system (2) × sonication (2)] were analyzed.

### 2.2. Gluten Sonication

Wet gluten was obtained according to the Polish Standard [16]. Gluten samples (2 mm thick) were put on the Petri dish floating on the surface of the ultrasonic scrubber. Samples were sonicated for 300 s in an ultrasonic scrubber Sonic-0.5 (POLSONIC Palczyński Sp. J., Warsaw, Poland) using 40 kHz ultrasound at 80 W. The effectiveness of the ultrasound was checked by observing holes in the aluminum foil caused by the cavitation.

### 2.3. Gluten Compression

A 2.5 g sample of gluten was weighed and gluten spheres were shaped manually. Gluten spheres were compressed using a TAXT2i texturometer (Stable Micro Systems, Haselemere, UK) to 70% deformation at the speed of 0.5 mm s^−1^. Six spheres were compressed for each gluten sample and the results were presented as the arithmetic mean.

### 2.4. Ultrasound Viscosity Measurements 

The probe was immersed into the gel and the values of viscosity x density (mPas × g cm^−3^) were measured. All the measurements were made using an ultrasound viscometer Unipan type 505 (UNIPAN, Warsaw, Poland). Six measurements were performed to obtain a single average result.

### 2.5. Dynamic Oscillatory Measurements 

Viscoelastic properties of gluten were measured using a Kinexus Lab+ dynamic rheometer (Malvern Instruments Limited, Malvern, UK). A serrated steel plate geometry (35 mm diameter, 2 mm gap size) was applied to limit the sliding effects. Oscillatory measurements with frequency sweeps in the range of 0.1–10 Hz at 2 °C were recorded. All the measurements were taken at a 5% strain, which was in the linear viscoelastic range determined formerly by the strain sweep.

### 2.6. Contact Angle Measurements 

Advancing and receding contact angles of ultrapure water (Milli-Q (18.2 mΩ) on the gluten surfaces were measured using the contact angle meter (GBX, Tallaght, Ireland). The contact angle meter was equipped with a digital camera and temperature/humidity-controlled measuring chamber. All measurements were conducted at 20 °C and 50% relative humidity and based on the sessile drop method with water [17]. A 6 µL droplet from a syringe was settled on the sample surface by means of an automatic deposition system. The advancing contact angle was evaluated from the droplet shape by the Win Drop++ software (GBX, Tallaght, Ireland). A 2 µL volume of the droplet was sucked back into the syringe and the receding contact angle was calculated from the computer program. The advancing and receding contact angles were measured for 10 droplets for each sample and the arithmetic mean was calculated.

### 2.7. Apparent Surface Free Energy

Equilibrium contact angles were calculated from the measurements of advancing and receding contact angles on the gluten samples. For this purpose, Equations (1)–(3) from Tadmor’s theory were used [18]:

(1) $$\Gamma_{a}\equiv {\left(\frac{sin^{3}\theta_{a}}{\left(2-3cos\theta_{a}+cos^{3}\theta_{a}\right)}\right)}^{1/3}$$

(2) $$\Gamma_{r}\equiv {\left(\frac{sin^{3}\theta_{r}}{\left(2-3cos\theta_{r}+cos^{3}\theta_{r}\right)}\right)}^{1/3}$$

(3) $$\theta_{Eq}=arccos\left(\frac{\Gamma_{a}\cos\theta_{a}+\Gamma_{r}cos\theta_{r}}{\Gamma_{a}+\Gamma_{r}}\right)$$

where *Γ_a_* and *Γ_r_* are the advancing and receding angle weight coefficients, respectively; *θ_a_*, *θ_r_*, and *θ_Eq_* are the advancing, receding, and equilibrium contact angles, respectively. The SFE of the solid surface, *γ_S_*, was calculated using the hysteresis approach proposed by Chibowski [19,20] and Chibowski and Terpiłowski [21] based on the contact angle hysteresis (CAH). Equation (4):

(4) $$\gamma_{s}=\frac{\gamma_{l}{\left(1+cos\theta_{a}\right)}^{2}}{2+cos\theta_{r}+cos\theta_{a}}$$

allows the determination of the ‘apparent surface free energy’, *γ_s_*, with *γ_l_*, the liquid surface tension. When the equilibrium contact angle is used for the calculation of *γ_s_*, Equation (4) transforms into:

(5) $$\gamma_{s}=\frac{1}{2}\gamma_{l}\left(1+\cos\theta_{Eq}\right)$$

Using this approach, the SFE was calculated from the wetting angles of water.

### 2.8. Gluten Surface Topography

The gluten surface was investigated using an optical profilometer GT Contour Surface Metrology (Veeco, Tucson, AZ, USA). The surface topography was characterized with very high accuracy in the range from the sub-nanometer to the 10 mm. Surface roughness was calculated using Vision 4.20 software (Veeco, Tucson, AZ, USA) [22].

### 2.9. Polarizing Optical Microscopy

The gluten surface microstructure was observed using a polarizing optical microscope Eclipse E600 Pol (Nikon, Tokyo, Japan).

### 2.10. Infrared Attenuated Total Reflectance (IR-ATR) Spectroscopy

IR-ATR spectra were obtained using a Nicolet iS10 FTIR spectrometer (ThermoFisher Scientific, Waltham, MA, USA). At the beginning of the research, the base spectrum was made. The samples were placed in the form of plates on the crystal because the change in surface chemical groups was the most interesting. The absorbance spectrum (4000–400 cm^−1^) was taken for each sample. The spectrum of a sample is based on 32 scans. Spectra processing and comparisons were made using OMNIC-9 software (ThermoFisher Scientific, Waltham, MA, USA).

### 2.11. Statistical Analysis

Tukey’s test was applied to determine statistically significant differences between means at *p* ≤ 0.05 and the Pearson correlation coefficients were calculated using the Statistica PL 13.3 program (TIBCO Software Inc., Palo Alto, CA, USA).

## 3. Results and Discussion

### 3.1. Protein Content in Wheat 

For all forecrops, the content of protein was higher for the organic farming system than the conventional one. For sugar beet and clover as forecrops, the difference was not statistically significant (Table 6). 

Similar findings were reported by Annet et al. [12], who noticed that organic farming produced a higher protein content in the grain than conventional farming did. Moreover, the content of protein reported was also significantly high for all farming systems and forecrops (>14%). In contracts, Baeckstrom et al. [13] noted that wheat grain from the conventional system had a higher protein concentration than the organic system. In this case, the protein concentrations for conventional and organic farming systems were 11.57 and 10.15, respectively. Interestingly, these contents were much lower than those obtained in this research (Table 6). If the protein content in wheat were high enough, the organic system would probably produce a higher protein concentration than the conventional system. 

For different forecrops, the highest concentration of protein was found in wheat grain cultivated after clover and sugar beet, while the lowest concentration was for wheat cultivated in monoculture. Wanic et al. [23] noted that the content of protein in wheat grain harvested after pea and oilseed rape was higher than in wheat cultivated in monoculture. Nemeiksiene et al. [24] found that for winter wheat, the protein content was higher when clover was used as a forecrop. Our research confirmed that cultivation in monoculture produced the lowest protein content in wheat grain regardless of the farming system. 

### 3.2. Rheological Properties of Gluten

**Figure 1:**
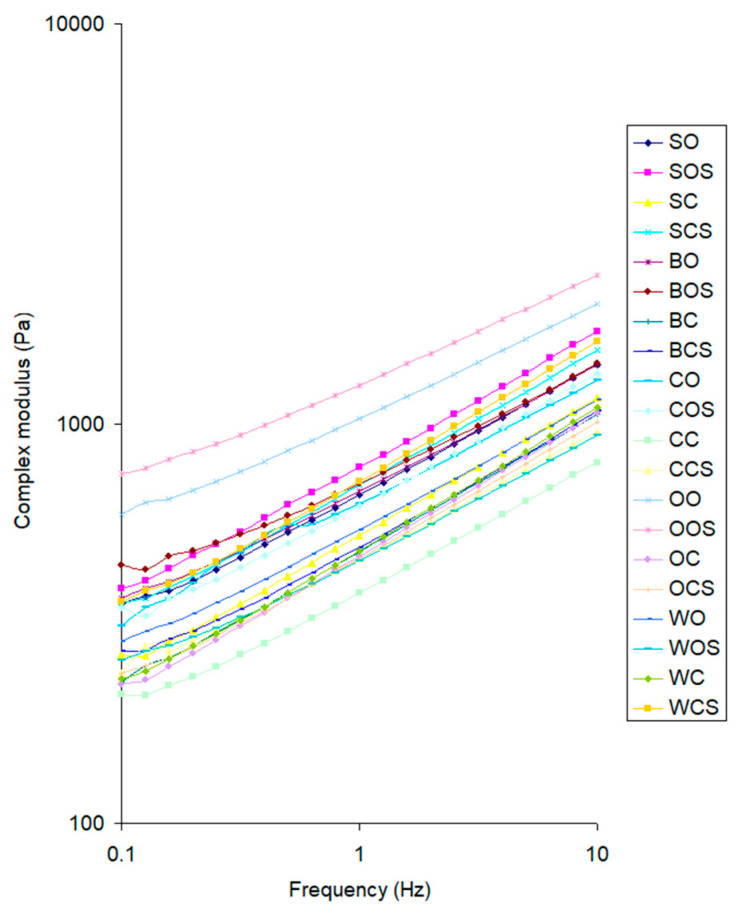
Influence of frequency on the complex modulus of different gluten samples (S—sugar beet; B—barley; C—clover; O—oats; W—wheat; O—organic system; C—conventional system; S—sonicated (e.g., BCS—barley in conventional system, sonicated).

Figure 1 shows the complex modulus of the samples measured at the frequency sweep test. 

There was an increase in the complex modulus value with the frequency. This was caused by the fact that the samples at higher frequency had less time for relaxation and behaved similar to a more solid material. Sonication increased the complex modulus value for most of the samples. Zhang et al. [25] investigated the effect of ultrasound on the rheological properties of gluten. Generally, the G′ and G″ of wheat gluten decreased by ultrasound treatment. It can be explained by the fact that in their research, gluten was dispersed into distilled water to form a homogeneous suspension. This suspension was ultrasound treated. In our research, we used pure gluten. Of ten samples with the highest values of complex modulus, only two were derived from the conventional system (Table 7). 

**Figure 2:**
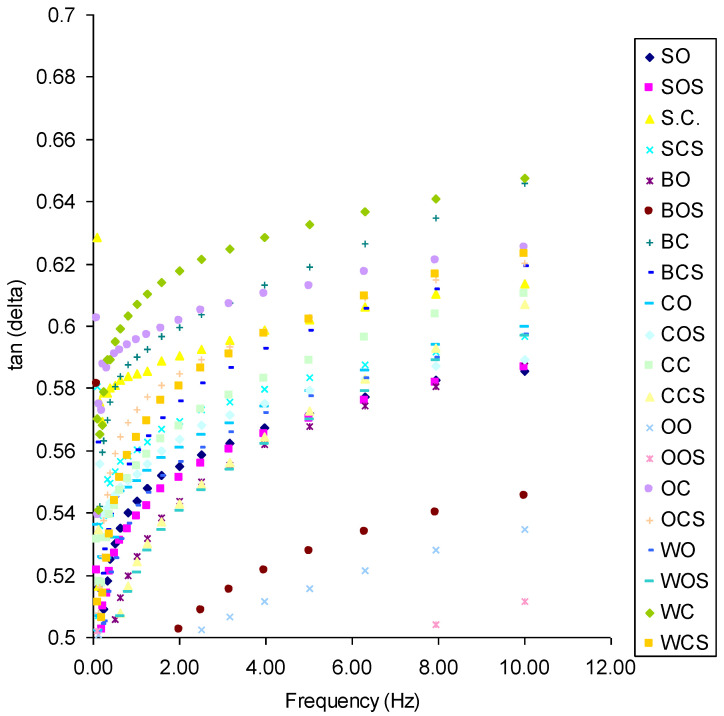
Influence of frequency on loss tangent of different gluten samples (S—sugar beet; B—barley; C—clover; O—oats; W—wheat; O—organic system; C—conventional system; S—sonicated (e.g., BCS—barley in conventional system, sonicated).

This is even more pronounced when we compare tangent delta values. There was an increase in the tangent delta value with the frequency (Figure 2).

Samples were more solid (higher G*, Figure 1) but less elastic (tan (delta) = G″/G′). The viscous element (G″) of the viscoelastic properties was higher at a higher frequency than the elastic element (G′). This can be explained by breaking some cross-links in gluten at a higher frequency when more energy was put into gluten at the same time. All values of tan(delta) for gluten from organically grown wheat were lower than those of conventionally grown wheat: both unsonicated and sonicated. Sonication also decreased the loss tangent values for all samples. The sonication effect was influenced by forecrops. The most elastic gluten after sonication was found for organic barley and sugar beet. The smallest values of tan(delta) were noted for conventional wheat and conventional oat (Figure 2). This showed that cultivation in the monoculture gave gluten with a smaller susceptibility to increase elasticity after sonic treatment. Wang et al. [26] and Lin and Cui [27] reported that excessive ultrasonic pre-treatment caused protein molecules to self-assemble, which may result in the increase in Young’s modulus of protein. In summary, organically grown wheat gluten is more elastic than conventionally grown wheat gluten and samples that are sonicated are more elastic than those that are not sonicated. More elastic samples are probably more cross-linked. A proof of this cross-linking is presented using infrared attenuated total reflectance (IR-ATR) spectroscopy.

Table 8 and Table 9 present the rheological properties (ultrasound viscosity and hardness in compression test, respectively) of gluten. 

We performed an analysis of the correlation between all investigated values and the coefficients of correlation are shown in Table 10. 

There was no correlation between any of the rheological properties and the content of protein in the grain. Gawęda et al. [28] investigated winter spelt cultivar ‘Rokosz’ and they found that gluten content, Zeleny sedimentation value, and dough development time increased with increasing protein content. Increased protein content caused gluten weakening to decrease, which increased the baking value [28]. We found a linear correlation between the complex modulus and ultrasound viscosity (R^2^ = 0.909), complex modulus and hardness (R^2^ = 0.889), and ultrasound viscosity and hardness (R^2^ = 0.823). The highest correlation was found between the complex modulus and ultrasound viscosity, which was probably caused by the fact that both are small-strain methods.

#### 3.2.1. Infrared Attenuated Total Reflectance (IR-ATR) Spectroscopy

**Figure 3:**
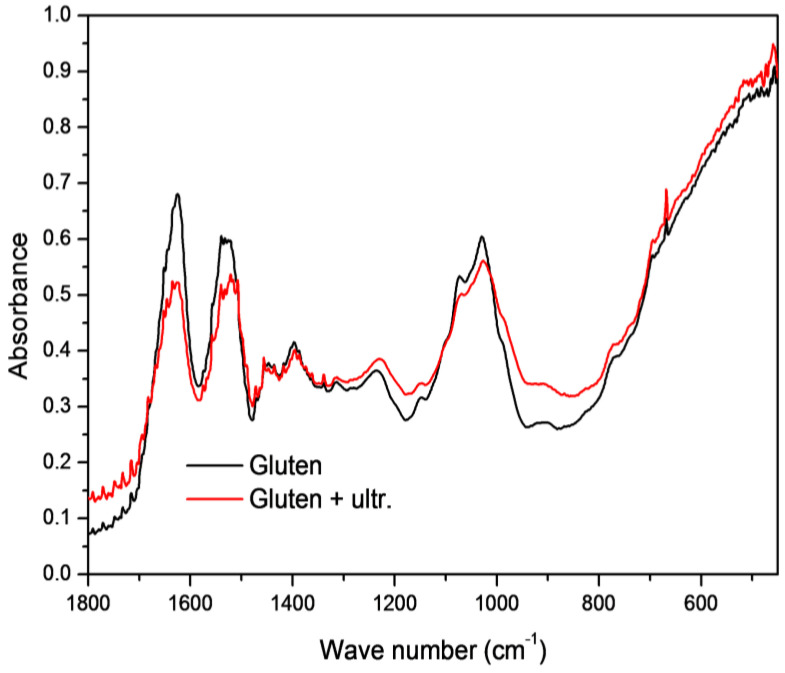
Raman spectra of unsonicated and sonicated gluten obtained from wheat cultivated in the organic system with barley as a forecrop.

Figure 3 shows a comparison of the Raman spectra of unsonicated and sonicated gluten obtained from wheat cultivated in the organic system with barley as a forecrop.

Similar differences between unsonicated and sonicated gluten samples were observed for all samples. A higher intensity was observed for sonicated gluten at 1230 cm^−1^. According to Nawrocka et al. [29], it shows stronger type I hydrogen bonds (–HN···O=C–) formed between polypeptide chains in the gluten network, leading to its aggregation. The β-sheet structures are visible at the frequencies of 1634–1640 and 1690–1692 cm^−1^ with the latter being typical for structures rich in intermolecular hydrogen bonds (antiparallel β-sheets, Aβ-sheet) [30]. For sonicated samples, a higher intensity is observed at 1690 cm^−1^, indicating the formation of hydrogen bonds at ultrasound processing. This would support the observed better rheological and textural properties of the sonicated samples. 

According to Sugeta [31], the spectral region from 490 to 550 cm^−1^ shows three conformations of disulfide bridges: *gauche-gauche-gauche* (g-g-g), *trans-gauche-gauche* (t-g-g), and *trans-gauche-trans* (t-g-t), which are assigned to the maxima at ca. 505, 520, and 530 cm^−1^, respectively. Additionally, the Raman spectra show two bands at ca. 515 and 540 cm^−1^ that are related to intrachain disulfide bridges [32]. In the whole region of 490–550 cm^−1^ for sonicated gluten, a higher absorbance was observed (Figure 3). This shows that the better rheological and textural properties of gluten after sonification can be explained by an increased number of disulfide bonds. Both the interchain disulfide bonds and hydrogen bonds mediated by glutamine side chains are crucial for stabilizing the gluten structure [33]. Tyrosine residues occur periodically throughout the length of the gluten protein with the bands at 830 and 850 cm^−1^. The ratio of these band intensities I(850)/I(830) (tyrosine doublet) is used as an indicator of hydrogen bonding of the phenolic hydroxyl groups. A decrease in the tyrosine doublet shows the burriedness of tyrosine residues with the formation of intramolecular hydrogen bonds, and an increase indicates exposition of the residues on the surface of the protein complex [34]. In our research, for sonicated samples, the tyrosine doublet was higher than for unsonicated gluten (0.96 and 0.89, respectively), which indicates exposition of the tyrosine residues on the surface of the protein complex.

In the case of gluten proteins, a change in the intensity of the band at 760 cm^−1^ gives information about the hydrophobicity of the indole ring [35]. A decrease in the band intensity indicates exposition of the tryptophan residues on the surface of the protein, whereas an increased 760 cm^−1^ band intensity shows burriedness of the tryptophan residues inside the hydrophobic environment of the protein. A higher intensity was noted for ultrasound-treated gluten at 760 cm^−1^, which indicates burriedness of the tryptophan inside the hydrophobic environment of the protein complex (Figure 3). An increase in the rheological properties of gluten after sonication could be partly caused by hydrophobic interactions. There was no regularity in spectra when comparing different farming systems and forecrops. 

#### 3.2.2. Contact Angle of Gluten Surface 

The water advancing contact angles (CA) of unsonicated and sonicated gluten samples are given in Table 11.

A hydrophilic surface nature (CA < 90°) was observed for all unsonicated samples. A significant influence of the farming system on the wetting properties of gluten was found. Most of the organically grown wheat samples showed a lower CA than the conventional ones. For instance, the CA for barley as a forecrop was 54.7° and 81.3° for organic and conventional systems, respectively. A similar trend was found for sugar beet, oat, or clover samples. Therefore, plant nutrition had the potential to change gluten surface characteristics [36]. Kwiatkowski et al. [37] found significant differences in the wheat amino acid content from the organic and conventional farming systems. The gluten properties are attributed to the amino acid composition and interactions. The surface wetting behavior depends on a delicate balance between polar and nonpolar amino acid side chains. The gluten proteins have a hydrophilic region surrounded by hydrophobic N- and C-terminal domains, whose global balance is more hydrophilic [38]. Nucia et al. [39] studied the wettability properties of 8 varieties of spring wheat conventionally grown. The authors reported hydrophilic characters for all gluten surfaces, and the CA ranged from 40° to 70°. Tunc et al. [40] also studied the wettability properties of wheat-gluten films. They reported a CA of 76°, which is similar to the one obtained in this study. The hydrophilic character was also attributed to the polar amino acids of the gluten protein. On the other hand, the lowest CA of 52.8° was found for the organic sugar beet, while the highest one was 85.8° for the organic wheat, indicating that the gluten wettability is also sensitive to the choice of forecrops (Table 11).

Concerning the ultrasound-treated samples, sonication led to large changes in the surface wettability of all gluten samples. Sonication promoted a more hydrophobic character, which was confirmed by an increment in CA. The highest CA increase was observed for the gluten surface derived from organic cultivation after sugar beet. In contrast, the lowest one was found for the gluten derived from conventional cultivation with barley as a forecrop. According to Abedi et al. [41], this trend might be attributed to the internal molecular arrangement between N- and C- terminal domains, which was disrupted by the mechanical stress of the sonication treatment. These terminal domains are the disorder propensity [42]. The cavitation led to perturbations in the gluten structure and the hydrophobic regions were gathered. An important feature of the hydrogen bonds is their ability to interchange under stress, allowing the re-orientation of proteins [36]. The Raman spectra indicated the formation of hydrogen bonds at ultrasound processing. The increase in hydrophobicity is a direct consequence of a higher noncovalent bonding character. These results are in agreement with similar studies on the surface hydrophobicity of corn gluten, whey protein, black bean protein, and wheat germ protein [43,44,45]. It is interesting to note that for all gluten samples, the influence of the sonication was greater for the organically grown samples than the conventional samples. The hydrophobic interactions in the protein complex, attributed to the higher intensity observed at 760 cm^−1^ for the sonicated samples in the Raman spectra, are also supported by the wetting study.

#### 3.2.3. Surface Free Energy (SFE) 

Complementary information on the wetting properties of a solid surface can be obtained from the SFE calculation. In the case of biomaterials, this value is useful because it allows for predicting interactions between the material surface and supportive layers. The SFE was calculated using the equilibrium CAs and the hysteresis approach, CAH [19]. The equilibrium CA was obtained from Tadmor’s equations [18]. The gluten surface free energy was negatively correlated with contact angle, where the coefficient of correlation was −0.922 (Table 10). This is a general rule: on a surface with poor wetting capability (low free energy), the liquid preserves more of the droplet shape. For a greater droplet shape, the contact angle is higher. A greater droplet shape means that the surface energy is weaker than the surface tension of the liquid.

Table 12 displays the gluten surface free energy as a function of the farming systems and forecrops before and after the ultrasound treatment.

It is shown that the total value of SFE decreased after the sonication treatment, regardless of the farming system and forecrops. The highest difference of 10.1 mJ/m^2^ was found for the conventional wheat sample. A decrease in the energy value means that the surface became more hydrophobic. An increment in the CA induced by the ultrasound treatment confirmed a decrease in the SFE and thus in the wettability of the given material. The SFE results from the intermolecular bonding interactions at an interface by van der Waals forces and hydrogen links between permanent and induced dipoles [21]. The SFE values suggest that the sonication promoted a different conformation in the gluten proteins where hydrophobic regions were more exposed at the interface. The noncovalent interactions intensified after the sonication, and played a key role in determining the post-treatment-increased hydrophobicity of the gluten surface. The main factor governing the wetting properties of gluten proteins may be the hydrophobic regions that enhanced the interaction between the protein and other molecules.

#### 3.2.4. Gluten Surface Topography 

The topography study complements the characterization of the surface as the contact angle strongly depends on the surface roughness. The quadratic mean of the surface roughness parameter R_q_ was selected for the topography study due to its higher sensitivity with respect to the other ones. The topography parameter of the gluten samples is given in Table 13.

**Figure 4:**
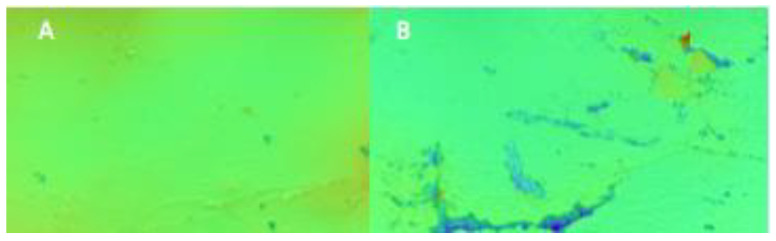
3D surface roughness profile of gluten obtained from wheat cultivated after sugar beet as a forecrop observed using optical profilometer. (**A**)—unsonicated; (**B**)—sonicated (surface = 0.9 × 1.3 mm).

Generally, the results obtained are consistent with those obtained for the CA measurements. It is clearly seen that the surface became rougher with the sonication, regardless of the farming system, which is manifested by an increment in the roughness parameter. Sonication treatment of gluten proteins rearranged the intermolecular linkages, especially disulfide and hydrophobic bonds, leading to changes in their surface morphology [11]. Figure 4 depicts the profilometer images obtained from sonicated and unsonicated wheat-gluten derived from sugar beet as a forecrop. The unsonicated samples showed a more homogeneous surface than the sonicated ones. The latter displayed hill and pit shapes distributed over the surface, which are typical for hydrophobic surfaces. A similar pattern was obtained for the other gluten samples (not shown). This type of surface hinders the drop spreading and, thus, the contact angle increases. 

**Figure 5:**
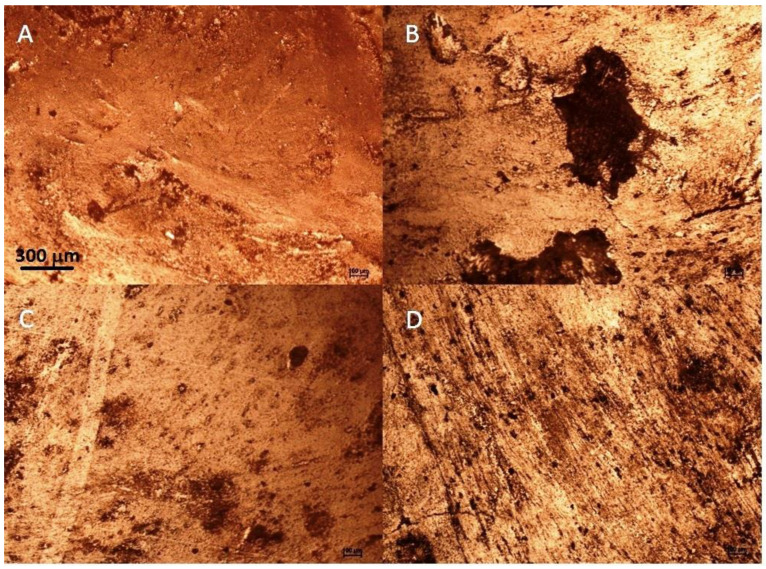
Microstructure of gluten obtained from wheat cultivated after barley as a forecrop observed using a polarizing optical microscope. (**A**)—organic unsonicated; (**B**)—organic sonicated; (**C**)—conventional unsonicated; (**D**)—conventional sonicated.

Zhang et al. [11] investigated the effect of ultrasound on wheat gluten surface roughness using different treatment times. The treatment was conducted from 0 to 25 min. They reported that as the treatment time increased to 15 min, the R_q_ greatly increased but decreased with longer treatment. Liang et al. [46] reported an increment in the roughness parameters after sonication of milk protein. The authors stated that the noncovalent interactions between protein molecules induced surface modification. Wang et al. [47] stated that the surface topography of wheat-gluten films increased after the sonication due to the formation of protein fragments on the surface. It has been elucidated that the wettability of the wheat-gluten is a direct consequence of its surface topography. The farming system and the sonication had a great influence on the surface properties of the wheat-gluten films. The sonication induced changes in the surface morphology, which were manifested by an increase in the surface roughness. Noted values of surface roughness are in agreement with the view of the gluten surface observed by polarizing optical microscopy. Figure 5 presents the surface of gluten obtained from the conventional and organic cultivation of spring wheat with barley as a forecrop. An increase in surface roughness after sonication of the samples from both cultivation methods can be easily seen.

A similar agreement between the roughness parameter and microscopic view of the surface could be seen for other samples with different cultivation methods and forecrops (not shown). According to Zhang et al. [48], the shear forces from ultrasound pretreatment disrupted the gluten bonds, thereby rupturing the structure. Further protein molecule aggregation could increase the surface roughness of gluten.

## 4. Conclusions

This study elucidated the potential for enhancing the spring wheat grain protein content by cultivation management strategies involving organic and conventional farming systems and the use of different forecrops. Moreover, the surface and rheological properties of wheat-gluten were upgraded by ultrasound treatment. The organic farming system produced a higher protein content than the conventional one, regardless of the used forecrop. Overall, the protein concentration found in the grains was high (13–15%), which is in agreement with the global trend of sustainable agricultural development aimed at increasing the production of plant proteins. Gluten derived from organic farming exhibited better rheological and surface properties. The wheat monoculture produced gluten with a lower susceptibility to increase elasticity after ultrasound treatment. The sonication of gluten proteins induced a rearrangement in the intermolecular linkages, especially disulfide and hydrophobic bonds, which led to changes in their surface morphology. Being able to tailor the surface and rheological properties of food byproducts can provide a wide range of unexplored possibilities in both food and nonfood applications. The prospect of organic farming is to achieve both higher protein yield and better technological value of different plant ingredients. The results obtained in this study indicate that environmentally friendly wheat cultivation methods are able to produce high protein content in the grain and gluten with enhanced technological potential.

## Figures and Tables

**Table 1:** Soil characteristics prior to establishing a spring wheat experiment (2017).

Farming Treatment	Soil pH 1 M KCl	N (%)	P mg kg^−1^	K mg kg^−1^	C Organic (%)
Organic	6.4	0.09	130	211	1.18
Conventional	6.3	0.13	135	219	1.26

**Table 2:** Spring wheat fertilization used in a conventional system.

Crop Plants	Mineral Fertilization (kg ha^−1^)		
	N	P	K
Spring wheat	70 (split doses) *	50 (before sowing)	90 (before sowing)

* N—70 kg (30 kg before sowing; 40 kg in spring at stem elongation—BBCH 32–34).

**Table 3:** Dates of sowing and harvesting of spring wheat forecrops.

Crop Plants	Sowing Date	Harvest Date
Sugar beet	19–23.04	17–20.10
Spring barley	19–22.04	11–13.08
Red clover	15–18.04	21–23.08
Winter wheat	22–25.09	10–12.08
Oat	12–15.04	19–21.08

**Table 4:** Fertilization of spring wheat forecrops used in the conventional system.

Crop Plants	Mineral Fertilization (kg ha^−1^)			Manure Fertilization (t ha^−1^)
	N	P	K	
Sugar beet	90 (before sowing)	90 (before sowing)	120 (before sowing)	25 (autumn; before sowing)
Spring barley	65 (before sowing)	45 (before sowing)	85 (before sowing)	-
Red clover	20 (before sowing)	30 (before sowing)	45 (before sowing)	-
Winter wheat	90 (before sowing)	70 (before sowing)	110 (before sowing)	-
Oat	45 (before sowing)	35 (before sowing)	55 (before sowing)	-

**Table 5:** Fertilization of spring wheat forecrops used in the organic system.

Crop Plants	Mineral Fertilization (Humac Agro) (kg ha^−1^)	Manure Fertilization (Originating from Organic Livestock Production) (t ha^−1^)
Sugar beet	450 (before sowing)	25 (autumn; before sowing)
Spring barley	320 (before sowing)	-
Red clover	55 (as top dressing)	-
Winter wheat	380 (before sowing)	-
Oat	280 (before sowing)	-

**Table 6:** Wheat grain protein content (%).

Spring Wheat Forecrops	Farming System	
	Organic	Conventional
S. Beet	15.23 ± 0.13 ^e^*	14.98 ± 0.14 ^e^
Barley	14.11 ±0.09 ^c^	12.87 ±0.19 ^a^
Clover	15.34 ±0.12 ^e^	15.02 ±0.10 ^e^
Oat	14.56 ±0.08 ^d^	13.44 ±0.22 ^b^
Wheat	14.04 ±0.09 ^c^	13.27 ± 0.16 ^b^

* Means with different letters (a–e) are significantly different (*p* ≤ 0.05).

**Table 7:** Complex modulus (Pa) of gluten samples.

Farming System	Spring Wheat Forecrops				
Organic	S. Beet	Barley	Clover	Oat	Wheat
Unsonicated	1409 ± 23 ^i^*	1405 ± 12 ^i^	1284 ± 16 ^h^	1987 ± 16 ^m^	1147 ± 31 ^fg^
Sonicated	1698 ± 11 ^l^	1410 ± 21 ^i^	1339 ± 18 ^h^	2352 ± 11 ^n^	933 ± 29 ^b^
Conventional	S. Beet	Barley	Clover	Oat	Wheat
Unsonicated	1163 ± 16 ^g^	1053 ± 31 ^de^	800 ± 13 ^a^	1060 ± 9 ^de^	1096 ± 23 ^ef^
Sonicated	1527 ± 21 ^j^	1077 ± 31 ^e^	953 ± 23 ^bc^	1009 ± 14 ^cd^	1608 ± 12 ^k^

* Means with different letters (a–n) are significantly different (*p* ≤ 0.05).

**Table 8:** Ultrasound viscosity (mPas g/cm^3^) of gluten samples.

Farming System	Spring Wheat Forecrops				
Organic	S. Beet	Barley	Clover	Oat	Wheat
Unsonicated	425 ± 12 ^c^*	500 ± 18 ^de^	470 ± 14 ^cd^	1170 ± 31 ^i^	320 ± 11 ^b^
Sonicated	1080 ± 22 ^h^	680 ± 14 ^f^	670 ± 6 ^f^	1250 ± 29 ^j^	300 ± 19 ^b^
Conventional	S. Beet	Barley	Clover	Oat	Wheat
Unsonicated	623 ± 31 ^e^	440 ± 9 ^c^	200 ± 10 ^a^	450 ± 11 ^cd^	420 ± 16 ^c^
Sonicated	790 ± 21 ^g^	680 ± 14 ^f^	540 ± 21 ^e^	420 ± 12 ^c^	1070 ± 28 ^h^

* Means with different letters (a–j) are significantly different (*p* ≤ 0.05).

**Table 9:** Hardness (N) of gluten samples.

Farming System	Spring Wheat Forecrops				
Organic	S. Beet	Barley	Clover	Oat	Wheat
R_q_ Unsonicated	2.24 ± 0.01 ^ef^*	2.29 ± 0.05 ^e–g^	2.02 ± 0.09 ^c–e^	2.56 ± 0.08 ^g^	1.59 ± 0.06 ^ab^
R_q_ Sonicated	2.47 ± 0.11 ^fg^	2.20 ± 0.01 ^d–f^	2.04 ± 0.07 ^c–e^	3.18 ± 0.06 ^h^	1.49 ± 0.09 ^a^
Conventional	S. Beet	Barley	Clover	Oat	Wheat
R_q_ Unsonicated	2.26 ± 0.10 ^ef^	1.92 ± 0.06 ^cd^	1.59 ± 0.05 ^ab^	1.54 ± 0.02 ^a^	1.87 ± 0.09 ^c^
R_q_ Sonicated	2.24 ± 0.01 ^ef^	2.19 ± 0.07 ^d–f^	1.86 ± 0.06 ^bc^	1.44 ± 0.06 ^a^	2.25 ± 0.12 ^ef^

* Means with different letters (a–h) are significantly different (*p* ≤ 0.05).

**Table 10:** Correlation coefficients between measured physicochemical properties of gluten and content of protein in the grain.

	Ultrasound Viscosity (mPas g cm^−3^)	G* in 10 Hz (Pa)	Hardness (N)	Protein in Grain (%)	Contact Angle	Surface Free Energy (mJ m^−2^)	Roughness R_q_
Ultrasound viscosity (mPas g cm^−3^)	1						
G* in 10 Hz (Pa)	0.909	1					
Hardness (N)	0.823	0.889	1				
Protein in grain P (%)	0.181	0.245	0.269	1			
Contact angle	0.057	−0.111	−0.160	−0.365	1		
Surface free energy (mJ m^−2^)	−0.115	−0.029	0.084	0.367	−0.922	1	
Roughness R_q_	−0.023	−0.071	0.050	0.055	−0.046	0.274	1

**Table 11:** Wheat gluten surface advancing contact angles (CA).

Farming System	Spring Wheat Forecrops				
Organic	S. Beet	Barley	Clover	Oat	Wheat
Unsonicated	52.80 ± 6.1 ^a^*	54.70 ± 5.2 ^a^	57.53 ± 3.3 ^ab^	60.76 ± 4.1 ^a–c^	85.88 ± 4.4 ^f–i^
Sonicated	74.73 ± 7.2 ^d–g^	72.93 ± 2.1 ^c–f^	75.44 ± 3.5 ^d–g^	77.18 ± 2.8 ^d–h^	94.00 ± 5.3 ^i^
Conventional	S. Beet	Barley	Clover	Oat	Wheat
Unsonicated	83.87 ± 2.1 ^e–i^	81.26 ± 2.4 ^e–i^	58.55 ± 5.0 ^ab^	74.00 ± 1.5 ^c–g^	63.53 ± 3.6 ^a–d^
Sonicated	90.83 ± 7.8 ^h–i^	87.23 ± 5.3 ^g–i^	70.60 ± 5.1 ^b–e^	84.90 ± 1.2 ^f–i^	2.1 ^e–i^

* Means with different letters (a–i) are significantly different (*p* ≤ 0.05).

**Table 12:** Wheat gluten surface free energy SFE (mJ m^−2^).

Farming System	Spring Wheat Forecrops				
Organic	S. Beet	Barley	Clover	Oat	Wheat
Unsonicated	60.3 ± 3.8 ^g–j^*	62.4 ± 2.7 ^ij^	61.3 ± 2.0 ^h–j^	55.9 ± 2.3 ^f–i^	44.9 ± 1.8 ^a–c^
Sonicated	51.6 ± 3.8 ^c–f^	55.8 ± 1.4 ^f–i^	53.9 ± 4.3 ^d–h^	52.8 ± 2.6 ^c–g^	39.3 ± 1.6 ^a^
Conventional	S. Beet	Barley	Clover	Oat	Wheat
Unsonicated	49.8 ± 1.5 ^b–f^	49.7 ± 0.8 ^b–f^	65.4 ± 5.2 ^j^	54.6 ± 1.0 ^e–i^	57.6 ± 2.2 ^f–j^
Sonicated	43.3 ± 1.1 ^ab^	46.5 ± 1.4 ^a–d^	61.9 ± 1.9 ^h–j^	50.0 ± 2.7 ^b–f^	47.5 ± 2.3 ^b–e^

* Means with different letters (a–j) are significantly different (*p* ≤ 0.05).

**Table 13:** Roughness parameter, R_q_ (µm), of wheat gluten surface (surface = 0.9 × 1.3 mm).

Farming System	Spring Wheat Forecrops				
Organic	S. Beet	Barley	Clover	Oat	Wheat
Unsonicated	0.63 ± 0.08 ^ab^*	0.90 ± 0.07 ^e^	0.64 ± 0.04 ^b^	0.68 ± 0.05 ^bc^	0.76 ± 0.08 ^bc^
Sonicated	0.75 ± 0.03^bc^	1.6 ± 0.08 ^h^	0.84 ± 0.03 ^cd^	0.96 ± 0.06 ^e^	0.75 ± 0.08 ^bc^
Conventional	S. Beet	Barley	Clover	Oat	Wheat
Unsonicated	0.51 ± 0.07 ^a^	0.81 ± 0.04 ^c^	1.14 ± 0.02 ^f^	0.69 ± 0.06 ^bc^	0.53 ± 0.02 ^a^
Sonicated	0.77 ± 0.06 ^c^	0.91 ± 0.03 ^e^	1.32 ± 0.06 ^g^	0.81 ± 0.06 ^cd^	0.70 ± 0.05 ^b^

* Means with different letters (a–h) are significantly different (*p* ≤ 0.05).

## Data Availability

The data supporting the results of this study are included in the manuscript.
